# COVID-19 vaccine hesitancy among university students in Lebanon

**DOI:** 10.1017/S0950268821002314

**Published:** 2021-11-02

**Authors:** M. Bou Hamdan, S. Singh, M. Polavarapu, T. R. Jordan, N. M. Melhem

**Affiliations:** 1 Medical Laboratory Sciences Program, Division of Health Professions, Faculty of Health Sciences, American University of Beirut, Beirut 1107 2020, Lebanon; 2 School of Population Health, College of Health and Human Services, The University of Toledo, 2801 W. Bancroft Street, Toledo, OH 43606, USA

**Keywords:** COVID-19 vaccine, hesitancy, Lebanon, resistance, university students

## Abstract

Little is known about the decision-making process of college students in Lebanon regarding coronavirus disease-2019 (COVID-19) vaccination. The aim of this study was to identify factors predicting behavioural intentions of students enrolled at the American University of Beirut to obtain a COVID-19 vaccine. A total of 3805 students were randomly selected. Participants were divided into three groups: vaccine accepting (willing to take or already took the vaccine), vaccine hesitant (hesitant to take the vaccine) and vaccine resistant (decided not to take the vaccine). Overall, participants were vaccine accepting (87%), with 10% and 3% being hesitant and resistant, respectively. Vaccine hesitancy was significantly associated with nationality, residency status and university rank. Participants who believed the vaccine was safe and in agreement with their personal views were less likely to be hesitant. Participants who did not receive the flu vaccine were more hesitant than those who did. Moreover, a significant association between hesitancy and agreement with conspiracies was observed. A high level of knowledge about COVID-19 disease and vaccine resulted in lower odds of vaccine resistance among students. The factors identified explaining each of the three vaccine intention groups can be used as core content for health communication and social marketing campaigns to increase the rate of COVID-19 vaccination.

## Background

On 11 March 2020, the World Health Organization (WHO) declared coronavirus disease-2019 (COVID-19) a global pandemic [1]. Severe acute respiratory syndrome coronavirus-2 (SARS-CoV-2), the causative agent of COVID-19 is a respiratory virus transmitted from person-to-person via droplets. With the lack of antiviral treatment and despite the implementation of non-pharmaceutical interventions (NPIs), the number of COVID-19 cases is still increasing worldwide [2–4]. Consequently, vaccines remain the most effective way to prevent the spread of COVID-19 [2, 5]. Currently, there are nine COVID-19 vaccines in early or limited use with eight vaccines approved for full use [2, 6–8]. The record time of development of these vaccines generated a global hesitancy among many and has affected the roll-out of vaccines to control the spread of SARS-CoV-2 [4].

*Vaccine hesitancy*, defined as the ‘delay in acceptance or refusal of vaccines despite availability of vaccine services’, has been an ongoing challenge [4, 9]. Vaccine hesitancy is caused by multiple factors and varies with time, place and vaccines [9, 10]. These factors include complacency (individual perceptions of the risks *vs.* the need for vaccination), convenience (availability, affordability and accessibility to vaccines) and confidence (trust in the safety and effectiveness of the vaccine and the delivering healthcare system and in the decisions of policymakers) [9]. The ‘3Cs’ are helpful in understanding factors that contribute to vaccine hesitancy [9, 11]. These include *contextual influences* (e.g. historic, socio-cultural, environmental, health system/institutional, economic or political factors), individual and group influences (personal perception of the vaccine including knowledge, awareness, conspiracy beliefs, attitudes or a personal experience with a vaccinated family member/friend), as well as vaccination influences (costs, mode of delivery, mode of administration, strength and knowledge of healthcare workers, risks or benefits) [12, 13].

The willingness to take the vaccine varies based on vaccine safety and effectiveness [14]. Several countries have conducted studies and reported on COVID-19 vaccine hesitancy. The highest COVID-19 vaccine acceptance rates were reported among adults in Ecuador (97%), Malaysia (94%), Indonesia (93%) and China (91%) [15–19]. High trust in government is posited as to why these acceptance rates are so high [20]. In the USA, vaccine acceptance rates were reported among adults (between 57.5% and 68.5%) [20], medical students (75.5%) [21], dental students (56%) [22] and the general population (78%) [23]. Unfortunately, COVID-19 vaccine acceptance rates in the Middle Eastern populations have been among the lowest worldwide: Lebanon (21%) [24], Jordan (28.4–37.4%) [11, 25, 26], Qatar (43%) [27], Iraq (62%) [5], Saudi Arabia (65%) [28], Turkey (66%) [29] and Israel (75%) [30].

University and college students form an important part of every society. Students are considered insightful, influential, open-minded, educated and responsive to public health issues [31]. Moreover, university students are considered as young highly knowledgeable adults who are at high risk of transmitting SARS-CoV-2 and at low risk of developing COVID-19-associated complications [32]. The general trend in research was to focus on vaccination perception among parents and physicians while neglecting university students. In the past few years, recent studies reported on vaccine hesitancy or acceptance among university students due to the shift in healthcare decision making from parents to university students [33, 34]. Moreover, the presence of gaps in knowledge about vaccine safety and effectiveness was reported among university students (e.g. nursing, medical and pharmacy students) [35]. To investigate COVID-19 vaccine hesitancy among this important population, various studies have been conducted in the region among medical students [4], dental students [14, 36] and university students in general [37, 38]. The purpose of this study was to identify the readiness, behavioural intentions and predictors of obtaining the COVID-19 vaccine among university students at the American University of Beirut (AUB). Our study is the first to use a theoretical approach to assess vaccine hesitancy among a large group of graduate and undergraduate students in Lebanon.

## Methods

### Study design, participants and sample size

The study was a randomised, cross-sectional study. It was approved by the Institutional Review Board (IRB) at the AUB. Participants were both undergraduate and graduate students enrolled at the AUB. At the time of the study, there were approximately 9495 students enrolled at the university: 7794 undergraduates and 1701 graduates. A sample size estimate using the sample size calculator from Raosoft (http://www.raosoft.com) indicated that 367 completed surveys from undergraduate students and 314 completed surveys from graduate students were needed to achieve external validity (95% confidence interval (CI); 5% margin of error and a 50% response distribution). Based on nearly an identical study in the USA that was completed just weeks before this one, we estimated a maximum of a 20% response rate. Therefore, we invited 3805 undergraduate and graduate students who were 18 years old and above to participate.

### Survey instrument

The survey instrument was built using LimeSurvey (LimeSurvey, version 3.14.8; Hamburg, Germany). Survey questions were developed based on previously published literature on attitudes and behaviours about vaccination [4, 21, 38]. The survey included questions based on the integrated behavioural model (IBM) (to assess attitude, perception and behavioural intentions) [39], precaution adoption process model (PAPM) (to assess readiness to get vaccinated) [40] and the extended parallel processing model (EPPM) (to assess cognition and emotional reactions) [41]. The survey was divided into sections with a total of 35 questions.

The first section asked questions related to the PAPM stage of readiness, history of COVID-19 infection and behavioural intentions towards getting the vaccine once available to students. The second section consisted of questions entailing participant's salience, instrumental and experiential attitudes and knowledge about SARS-CoV-2 vaccine. Salience was assessed using three questions to determine the importance of getting vaccinated among students (4-point scale ranging from ‘not important at all’ to ‘very important’). Instrumental and experiential attitudes were assessed using seven questions to determine the level of favourability among students towards the COVID-19 vaccine (5-point scale with 5 being the most favourable). Knowledge about SARS-CoV-2 infection and the vaccine was assessed using eight true/false questions. The third section of the online survey included eight questions that assessed students' level of confidence to perform actions related to getting a COVID-19 vaccine (4-point scale questions from ‘not confident at all’ to ‘very confident’). The fourth section included questions related to environmental constraints and perceived behavioural control towards vaccination. Environmental constraints were assessed using 12 questions related to the effect of specific environmental conditions on getting or not getting the vaccine (a 5-point scale from ‘very easy’ to ‘very difficult’ and a 4-point scale from ‘significant impact’ to ‘no impact’). Perceived control of getting the COVID-19 vaccine was assessed in a 5-point scaled question (from ‘not under my control’ to ‘completely under my control’).

The fifth section of the survey assessed perceived social norms (descriptive and subjective norms). Descriptive norms were assessed using eight questions related to the likelihood of individuals in the student's social network to get the COVID-19 vaccine (5-point scale ranging from ‘very unlikely’ to ‘very likely’ in addition to ‘not applicable’). Subjective norms were measured using eight questions related to the perceived influence of others regarding obtaining the vaccine (4-point scale ranging from ‘not influential at all’ to ‘very influential,’ in addition to ‘not applicable’).

Finally, section six of the survey targeted conspiracy beliefs regarding the importance, safety and efficacy of the vaccine in addition to the financial aspects of the vaccine. These included beliefs related to the media as a driver of unnecessary fear about COVID-19, the potential harm from the vaccine *vs.* COVID-19 disease, the more severe impact of flu compared to COVID-19, the hidden information by pharmaceutical companies regarding vaccination health outcomes and the vaccine as an attempt to control people and take away personal freedom. The last section addressed socio-demographic characteristics of students (age, gender, programme of study, rank and nationality), health status (flu vaccination habit and history of chronic diseases) and sources of knowledge about COVID-19 (social media, friends, family, healthcare workers, YouTube, TV, medical journals, government websites and medical websites).

### Data collection

Data were anonymously collected between 11 May 2021 and 18 June 2021 – the date marking the start of the vaccination campaign at the AUB. The registrar's office at the AUB provided the IRB with a random list of undergraduate and graduate students who previously agreed to share their contact information. Consequently, the IT team, in collaboration with the IRB, uploaded the emails of those students to LimeSurvey. Invitations and reminders (*n* = 4) were sent anonymously to potential participants via standardised mass emails containing the eligibility criteria and the survey link. The investigators did not request or have any location on the survey where students could mark their identity or any identifying information. The survey link was also shared on official university platforms on social media (Facebook Inc., Twitter, Instagram and Whatsapp messenger). The survey was accessible to undergraduate and graduate students who are ≥18 years of age and enrolled at the AUB.

### Statistical analysis

Data analysis was performed using STATA SE 13.0. Descriptive statistics (frequencies, percentages and means) were reported. We generated three groups to categorise vaccine willingness: (1) vaccine accepting (those who are willing to take or already took the vaccine), (2) vaccine hesitant (those who are hesitant to take the vaccine) and (3) vaccine resistant (those who decided not to take the vaccine). Chi-square test of proportions was used to compare categorical variables across the vaccine willingness groups (accepting, hesitant and resistant). Backward stepwise regression and multiple logistic regression models were used to assess the association between the vaccine hesitancy or resistant groups and their knowledge about COVID-19 and its vaccine, environmental constraints, self-efficacy, perceived control, instrumental and experiential attitudes, salience, response cost and efficacy and descriptive and subjective norms. We simultaneously estimated the odds ratios (ORs) with its corresponding 95% CIs to determine the associations between the variables retained from the stepwise regression (*P* <0.05) and the hesitancy group (comparison group) *vs.* vaccine acceptance group (reference group). Similarly, we estimated the OR and its 95% CI to determine the associations between the retained variables and the vaccine resistant group (comparison group) *vs.* its reference group (hesitancy and acceptance groups). We adjusted for control variables (views regarding the impact of COVID-19 on Lebanon, views regarding getting vaccinated against COVID-19, conspiracy thinking, flu vaccination habit and health behaviours) in both multivariable logistic regression models. A *p*-value <0.05 was considered statistically significant.

## Results

### Demographic characteristics of study participants

Of the 3805 students who were invited to participate, 800 participants (21%) from seven academic units at the AUB completed the survey. The mean age was 21 ± 0.14 years with the majority being Lebanese (85%), undergraduate students (75%) and females (57%) (Table 1). The acceptance, hesitant and resistant groups represented 87%, 10% and 3% of students enrolled in the study, respectively. Our data show that residency area, nationality and university rank differed significantly among the three groups (Table 1).

**Table 1.** Demographic characteristics of study participants and COVID-19 vaccine intentions

	Respondents *N* (%)	Accepting (%)	Hesitant (%)	Resistant (%)	*P* value
Age (*N* = 724)					
⩽20 years	416 (57)	358 (57)	45 (61)	13 (620)	
21–30 years	280 (39)	247 (39)	26 (35)	7 (33)	
31–40 years	28 (4)	24 (4)	3 (4)	1 (5)	
Total		629	74	21	0.944
Gender (*N* = 758)					
Female	433 (57)	372 (56.5)	49 (62)	12 (54.5)	
Male	325 (43)	285 (43.5)	30 (38)	10 (45.5)	
Total		657	79	22	0.901
Nationality (*N* = 757)					
Lebanese	646 (85)	571 (87)	58 (75.5)	17 (77.5)	
Syrian	23 (3)	18 (2.5)	4 (5)	1 (4.5)	
Palestinian	25 (3.5)	23 (3.5)	1 (1.5)	1 (4.5)	
Other	63 (8.5)	46 (7)	14 (18)	3 (13.5)	
Total		658	77	22	**0**.**020**
University rank (*N* = 759)					
Freshmen	27 (4)	20 (3)	6 (7.5)	1 (4.5)	
Sophomore	175 (23)	140 (21)	26 (33.5)	9 (41)	
Junior	180 (23.5)	157 (24)	19 (24.5)	4 (18)	
Senior	186 (24.5)	173 (26.5)	11 (14)	2 (9)	
Graduate student	191 (25)	169 (25.5)	16 (20.5)	6 (27.5)	
Total		659	78	22	**0**.**012**
Residency status (*N* = 754)					
Local students	669 (89)	589 (90)	62 (78.5)	18 (82)	
International students	85 (11)	64 (10)	17 (21.5)	4 (18)	
Total		653	79	22	**0**.**005**
Living status (*N* = 763)					
Alone	46 (6)	36 (5.5)	8 (10)	2 (9)	
With others	717 (94)	623 (94.5)	71 (90)	20 (91)	
Total		659	79	22	0.216
Age of people sharing same housing (*N* = 763)					
<18 years	272 (36)	236 (36)	28 (35.5)	8 (36.5)	0.953
19–40 years	390 (51)	338 (51.5)	40 (50.5)	12 (54.5)	0.839
41–65 years	602 (79)	527 (80)	58 (73.5)	17 (77.5)	0.814
⩾66 years	133 (17)	120 (18)	10 (12.5)	3 (13.5)	0.521
Total		659	79	22	
Area of residence (*N* = 793)					
Beirut	369 (46)	325 (47)	37 (45.5)	7 (32)	0.363
Beqaa	24 (3)	20 (3)	2 (2.5)	2 (9)	0.237
Mount Lebanon	291 (36)	256 (37)	29 (36)	6 (27.5)	0.632
Nabatiyeh	17 (2)	15 (2)	1 (1.5)	1 (4.5)	0.629
North Lebanon	38 (5)	29 (4)	6 (7.5)	3 (13.5)	0.064
South Lebanon	48 (6)	41 (6)	4 (5)	3 (13.5)	0.299
Total		690	81	22	

*χ*^2^ tests were used to compare the frequencies in the cells for each variable. A *p*-value of <0.05 was considered significant.

### Health behaviours, perceptions and COVID-19 vaccines

We assessed the variation of health status and health behaviours among the vaccine groups: accepting, hesitant and resistant groups. Our data show that the three groups differed significantly on several variables including adherence to NPIs (wearing a facemask, social distancing, hand hygiene, avoiding in-door spaces and going out) as well as a history of flu vaccination (Table 2). It was clear that almost all respondents who received the flu vaccine during the past 3 years were accepting of the COVID-19 vaccine. Our data show that previous history of COVID-19 infection and testing positive for COVID-19 did not differ among the three vaccine willingness categories (Table 2). Similarly, these groups significantly differed when we enquired about an agreement with conspiracy beliefs and perceptions about COVID-19 infection and vaccine (Table 3). Pharmaceutical companies, manipulation by a higher power, the vaccine taking away personal freedom and governmental control were mainly where the three groups differed.

**Table 2.** Health behaviours and acceptability of COVID-19 vaccine among study participants

	Respondents *N* (%)	Accepting (%)	Hesitant (%)	Resistant (%)	*P* value
COVID-19 infection history (*N* = 730)					
Yes	200 (27)	171 (26.5)	22 (32)	7 (35)	
No	530 (73)	470 (73.5)	47 (68)	13 (65)	
Total		641	69	20	0.200
Tested for COVID-19 (*N* = 792)					
Yes	533 (67)	467 (68)	52 (64)	13 (59)	
No	259 (33)	221 (32)	29 (36)	9 (41)	
Total		688	81	22	0.864
Tested positive for COVID-19 (*N* = 685)					
Yes	200 (29)	171 (28.5)	22 (33.5)	7 (35)	
No	485 (71)	427 (71.5)	44 (66.5)	13 (65)	
Total		598	66	20	0.886
COVID-19 infection history among people in the same social circle (*N* = 788)					
Yes	776 (98)	679 (99)	75 (95)	21 (95.5)	
No	12 (2)	7 (1)	4 (5)	1 (4.5)	
Total		686	79	22	**0**.**039**
Hospitalisation due to COVID-19 among people in the same social circle (*N* = 754)					
Yes	587 (78)	522 (79)	50 (68.5)	15 (68)	
No	167 (22)	137 (21)	23 (31.5)	7 (32)	
Total		659	73	22	**0**.**019**
Death due to COVID-19 among people in the same social circle (*N* = 763)					
Yes	495 (65)	438 (65.5)	42 (57)	14 (66.5)	
No	268 (35)	229 (34.5)	32 (43)	7 (33.5)	
Total		667	74	21	0.085
Flu vaccination history in the past few years (*N* = 712)					
3 times	91 (13)	87 (14.5)	2 (2.5)	2 (9)	
1 time	62 (9)	56 (9)	5 (6.5)	1 (4.5)	
None	353 (49)	299 (49)	34 (43.5)	18 (82)	
Unsure/don't remember	206 (29)	167 (27.5)	37 (47.5)	1 (4.5)	
Total		609	78	22	**<0**.**001**
*Health behaviour during the past month*					
Face mask (*N* = 742)					
All or most of the time	638 (86)	569 (88)	54 (73)	14 (63.5)	
Some of the time	92 (12)	67 (10.5)	19 (25.5)	6 (27.5)	
Never	12 (2)	9 (1.5)	1 (1.5)	2 (9)	
Total		645	74	22	**<0**.**001**
Social distancing of minimum 6 ft (*N* = 752)					
All or most of the time	322 (43)	287 (44)	25 (32.5)	9 (41)	
Some of the time	350 (46.5)	304 (47)	37 (48)	9 (41)	
Hardly ever	64 (8.5)	50 (7.5)	12 (15.5)	1 (4.5)	
Never	16 (2)	9 (1.5)	3 (4)	3 (13.5)	
Total		650	77	22	**0**.**001**
Washing hands (*N* = 746)					
All or most of the time	618 (83)	549 (85)	54 (70)	15 (68)	
Some of the time	114 (15)	88 (13.5)	20 (26)	6 (27.5)	
Hardly ever	14 (2)	10 (1.5)	3 (4)	1 (4.5)	
Total		647	77	22	**0**.**025**
Avoiding indoor spaces (*N* = 747)					
All or most of the time	357 (48)	320 (49.5)	27 (35)	10 (45.5)	
Some of the time	306 (41)	264 (40.5)	38 (49.5)	4 (18)	
Hardly ever	66 (88)	52 (8)	9 (11.5)	5 (23)	
Never	18 (3)	12 (2)	3 (4)	3 (13.5)	
Total		648	77	22	**0**.**001**
Avoiding close contact with COVID-19 positive cases (*N* = 724)					
All or most of the time	665 (92)	585 (93)	62 (86)	18 (82)	
Some of the time	59 (8)	45 (7)	10 (14)	4 (18)	
Total		630	72	22	**0**.**020**
Intentionally not going out (*N* = 708)					
All or most of the time	488 (69)	441 (71.5)	39 (55.5)	8 (38)	
Some of the time	195 (27.5)	159 (26)	28 (40)	8 (38)	
Never	25 (3.5)	17 (2.5)	3 (4.5)	5 (24)	
Total		617	70	21	**<0**.**001**
Physical health status (*N* = 754)					
Excellent/good	363 (48)	319 (48.5)	34 (43.5)	10 (45.5)	
Fair	17 (2)	15 (2.5)	1 (1.5)	1 (4.5)	
Poor	377 (50)	323 (49)	43 (55)	11 (50)	
Total		657	78	22	0.202
Mental health status (*N* = 757)					
Excellent/good	378 (50)	331 (50.5)	38 (49)	9 (41)	
Fair	102 (13.5)	91 (14)	9 (11.5)	2 (9)	
Poor	277 (36.5)	235 (35.5)	31 (39.5)	11 (50)	
Total		657	78	22	0.312
Needed a health care provider in the past 12 months for physical health (*N* = 754)					
Yes	359 (48)	315 (48.5)	34 (43)	10 (45.5)	
No	344 (46)	298 (45.5)	37 (47)	9 (41)	
Unsure	51 (6)	40 (6)	8 (10)	3 (13.5)	
Total		653	79	22	0.433
Needed a health care provider in the past 12 months for mental health (*N* = 754)					
Yes	136 (18)	122 (18.5)	11 (14)	3 (13.5)	
No	580 (77)	502 (77)	62 (78.5)	16 (73)	
Unsure	38 (5)	29 (4.5)	6 (7.5)	3 (13.5)	
Total		653	79	22	0.205

*χ*^2^ tests were used to compare the frequencies in the cells for each variable. A *p*-value of <0.05 was considered significant.

**Table 3.** Conspiracies and perceptions about COVID-19 infections and vaccines among study participants

	Respondents *N* (%)	Accepting (%)	Hesitant (%)	Resistant (%)	*P* value
Impact of COVID-19 on Lebanon (*N* = 790)					
Behind us	360 (46)	324 (47)	28 (35)	8 (36.5)	
Happening currently	221 (28)	189 (27.5)	24 (30)	8 (36.5)	
Still to come (in the future)	209 (26)	175 (25.5)	28 (35)	6 (27)	
Total		688	80	22	0.204
Views regarding COVID-19 vaccination (*N* = 757)					
Personal choice	103 (14)	61 (9)	27 (38.5)	15 (71.5)	
Everyone's responsibility	363 (48)	349 (52.5)	13 (18.5)	1 (4.5)	
Both	291 (38)	256 (38.5)	30 (43)	5 (24)	
Total		666	70	21	**<0**.**001**
Mainstream media creating unnecessary fear about COVID-19 (*N* = 757)					
Disagree	156 (20.5)	142 (21.5)	10 (12.5)	3 (13.5)	
Agree	401 (53)	345 (53)	43 (54.5)	13 (59)	
Neither	200 (26.5)	166 (25.5)	26 (33)	6 (27.5)	
Total		653	79	22	**0**.**011**
COVID-19 vaccine is not needed (*N* = 755)					
Disagree	639 (85)	579 (89)	52 (66)	5 (22.5)	
Agree	34 (14)	19 (3)	6 (7.5)	9 (41)	
Neither	82 (11)	53 (8)	21 (26.5)	8 (36.5)	
Total		651	79	22	**<0**.**001**
Potential harm of COVID-19 infection is exaggerated (*N* = 753)					
Disagree	437 (58)	402 (62)	26 (33)	6 (28.5)	
Agree	136 (18)	103 (16)	21 (26.5)	12 (57)	
Neither	180 (24)	145 (22)	32 (40.5)	3 (14.5)	
Total		650	79	21	**<0**.**001**
Death/year from flu are millions more than that of COVID-19 (*N* = 749)					
Disagree	189 (25)	167 (26)	13 (16.5)	9 (43)	
Agree	230 (31)	189 (29)	32 (40.5)	7 (33)	
Neither	330 (44)	290 (45)	34 (43)	5 (24)	
Total		646	79	21	**0**.**003**
COVID-19 vaccine is more dangerous than the disease itself (*N* = 752)					
Disagree	595 (79)	551 (85)	36 (45.5)	5 (23)	
Agree	35 (5)	20 (3)	6 (7.5)	9 (41)	
Neither	122 (16)	77 (12)	37 (47)	8 (36)	
Total		648	79	22	**<0**.**001**
Pharmaceutical companies have hidden information about the vaccine's bad health outcomes (*N* = 752)					
Disagree	414 (55)	388 (60)	22 (28)	3 (13.5)	
Agree	76 (10)	46 (7)	17 (21.5)	12 (54.5)	
Neither	262 (35)	214 (33)	40 (50.5)	7 (32)	
Total		648	79	22	**<0**.**001**
No sense to get the vaccine, higher power manipulates health outcomes (*N* = 750)					
Disagree	587 (78)	538 (83)	39 (50)	7 (33.5)	
Agree	39 (5)	22 (3.5)	7 (9)	10 (47.5)	
Neither	124 (17)	88 (13.5)	32 (41)	4 (19)	
Total		648	78	21	**<0**.**001**
COVID-19 vaccine is an attempt to take away personal freedom (*N* = 749)					
Disagree	610 (81)	553 (85.5)	50 (65)	4 (19)	
Agree	43 (6)	23 (3.5)	8 (10.5)	12 (57)	
Neither	96 (13)	72 (11)	19 (24.5)	5 (24)	
Total		648	77	21	**<0**.**001**
Government is using the vaccine to control population (*N* = 751)					
Disagree	564 (75)	517 (79.5)	39 (50)	5 (24)	
Agree	58 (8)	35 (5.5)	12 (15.5)	11 (52)	
Neither	129 (17)	97 (15)	27 (34.5)	5 (24)	
Total		649	78	21	**0**.**027**

*χ*^2^ tests were used to compare the frequencies in the cells for each variable. A *p*-value of <0.05 was considered significant.

### Factors associated with vaccine hesitancy and resistance among AUB students

To determine the factors that predicted vaccine hesitancy and resistance, we performed a backward stepwise regression and a multivariate logistic regression using the IBM construct variables while adjusting for confounders. The IBM variables include knowledge (outside the model) descriptive norm, subjective norm, environmental constraints (outside the model), self-efficacy, perceived control, salience (outside the model), response cost and efficacy and instrumental and experiential attitudes. The confounders included variables related to conspiracy theories, getting the flu vaccine, views towards getting the vaccine, views towards the status of the pandemic in Lebanon and personal health behaviours related to COVID-19 prevention.

Disagreement with the statement that symptomatic cases are the only carriers of SARS-CoV-2 was a statistically significant predictor of hesitancy (OR = 5; 95% CI = 1.67–14.29; *P* = 0.004). However, participants who felt that the vaccine was safe (OR = 0.01; 95% CI = 0.002–0.08; *P* <0.001) and was in agreement with their personal views (OR = 0.1; 95% CI = 0.02–0.51; *P* = 0.004) despite the discouraging stories about the vaccine (OR = 0.1; 95% CI = 0.01–0.57; *P* = 0.01) were less likely to be hesitant (Table 4A).

**Table 4.** Factors associated with hesitancy towards COVID-19 vaccine among AUB students

	OR	95% CI	*P* value
*(A) Hesitant group*			
Importance of getting the COVID-19 vaccine			
Not important	Ref		
Important	1 (omitted)		
Importance of decreasing odds of dying from COVID-19			
Not important	Ref		
Important	0.100	0.030–0.690	**0**.**015**
Feelings towards getting the vaccine: opposed to views *vs*. in agreement with views			
Opposed to views	Ref		
In agreement with views	0.100	0.020–0.510	**0**.**004**
Only symptomatic cases spread COVID-19			
True	Ref		
False	5	1.670–14.290	**0**.**004**
Vaccine safety wasn't adequately tested by pharmaceutical companies			
True	Ref		
False	0.500	0.190–1.43	0.210
Confidence about paying the cost of vaccines			
Not confident	Ref		
Confident	2	0.440–6.590	0.439
Confidence about getting one or more vaccine shots as recommended by health professionals			
Not confident	Ref		
Confident	0.010	0.002–0.080	**<0**.**001**
Difficulty of taking a time off from school/work on getting the vaccine			
Difficult	Ref		
Easy	2	0.530–7.950	0.297
Difficulty of hearing/reading things from others that discourage people from getting the vaccine			
Difficult	Ref		
Easy	0.100	0.010–0.570	**0**.**010**
Model statistics: adjusted *R*^2^ = 0.285, *F*_(9, 584)_ = 50.680), *P* < 0.001			
*(B) Resistant group*			
Likelihood that immediate family members already received or will receive the COVID-19 vaccine			
Unlikely	Ref		
Likely	2	0.120–39.580	0.585
Vaccine safety wasn't adequately tested by pharmaceutical companies			
True	Ref		
False	0.040	0.001–1.160	0.062
Vaccines use a new technology that changes the DNA of cells			
True	Ref		
False	14	0.290–678.090	0.181
Model statistics: adjusted *R*^2^ = 0.077, *F*_(3, 716)_ = 21.130, *P* < 0.001			

^a^Backward stepwise regression and multiple logistic regression models were used to assess the association between the vaccine hesitant or resistant groups and multiple variables while adjusting for controls. A *P* <0.05 was considered statistically significant.

Following adjustment for confounders while retaining the variables from the backward stepwise regression with *P* <0.05, our data showed that none of these variables was significantly associated with vaccine resistance as compared to control groups (accepting and hesitant groups) (Table 4B).

## Discussion

The WHO listed vaccine hesitancy as a top 10 threat to the control of vaccine-preventable diseases [42]. Vaccine hesitancy is usually linked to ideological beliefs as well as conspiracy ideations [43]. University students are a core part of society and a critical demographic to vaccination decision-making. During the college years is when most young adults become independent and responsible for their own health decision-making. University students are also leaders and play a critical role in spreading positive and informed facts about vaccines, thus influencing future generations [38]. Consequently, identifying the willingness of university students to take the COVID-19 vaccine and the factors that predict vaccine hesitancy are important to the development of effective health communication campaigns and strategies to increase the rate of vaccination.

Few studies reported vaccine acceptability among university students in this region of the world. Our main findings show that the COVID-19 vaccine acceptance was high (87%) among students enrolled at the AUB and much higher than university students in Jordan (35%) [38], dental students in Palestine (58%) [14], university students and staff in Qatar (62.5%) [37] and medical students in Egypt (35%) [4]. However, our results were similar to those reported among university students in Italy in terms of willingness to take the vaccine (86%) [31] and higher than those reported in France (58%) [44].

It was interesting to note that our findings showed that males had higher intentions to obtain the vaccine than females. This result was corroborated by previous studies conducted among adults and dental students in Lebanon [24, 36] and among university students in Jordan [38]. Moreover, Lebanese students had higher intentions to get the vaccine compared to other nationalities. Interestingly, a study of university students in Jordan reported that non-Jordanians had higher intentions to get vaccinated [38]. Further research is needed to shed more light on this apparent difference by nationality.

The impact of receiving a past influenza vaccine was also influential. Our results showed that vaccine willingness/acceptability differed significantly by students’ flu vaccination habits of the recent past. Those who did not take the flu vaccine in the past 3 years were more vaccine hesitant than those who did. Our results were corroborated by the results of previous studies that showed that those who did not take the flu vaccine in 2019 were also less likely to take the COVID-19 vaccine [14, 45, 46].

Numerous conspiracy theories regarding SARS-CoV-2 infection, the pandemic and COVID-19 vaccines have been and continue to be widely spread on social media. It is important to keep in mind that this is the first pandemic the world has experienced with social media. Thus, not only do we have a pandemic, but also we have an *infodemic* [47]. Misinformation and conspiracy theories are spread quickly among those who do not know how to evaluate the veracity of information. The current infodemic points to a need for a comprehensive, global approach to creating valid, reliable and trustworthy health information [47]. These conspiracy theories have included that the virus was man-made, was caused by 5G cell phone towers, that the vaccine injects microchips to manipulate humans, changes human DNA and causes infertility among females [4, 11, 48]. Our findings show the significant impact of conspiracy beliefs among the vaccine willingness groups. However, our results were contradictory to a previous study reporting an independent correlation between hesitancy and conspiracy beliefs among university students in Jordan [38]. Previous studies reported that the lack of accurate information about vaccine safety, effectiveness and side effects in addition to misinformation from social media played a major role in hesitancy [4, 14, 20, 36].

In its recent report, the WHO advanced three strategies to increase COVID-19 vaccine acceptance: harnessing social influences, increasing motivation through transparent risk communication strategies and creating an enabling environment through making vaccination accessible and affordable [49]. Each of these strategies can be used by countries in our region of the world. Our results also show that higher salience and positive instrumental (cognitive beliefs about outcomes of vaccines) and experiential attitudes (emotional responses to the vaccination) in relation to vaccine safety, efficacy and personal views towards vaccination resulted in lower odds of vaccine hesitancy. Moreover, higher self-efficacy except for those being confident about paying the cost of vaccines was associated with lower odds of vaccine hesitancy. Furthermore, a high level of knowledge about COVID-19 disease and vaccine resulted in lower odds of vaccine resistance among AUB students. However, higher descriptive norms were associated with higher odds of vaccine resistance. Similar results were reported in previous studies among university and dental students in the region [14, 36].

The results of this theory-based study can be utilised to design and test educational messaging campaigns to promote vaccination. However, our results should be cautiously interpreted. First, with a 20% response rate, a non-response bias may have been at work. Those students who did not respond may have affected the aggregated group results had they completed the survey. Second, although the study body at the AUB is diverse, we only surveyed students at the AUB. Therefore, the results are not generalizable to students at other universities in Lebanon or elsewhere. Moreover, this study was carried out before the vaccination campaign at the AUB and opinions may have changed since then.

## Conclusion

The widespread prevalence of SARS-CoV-2 and its variants, and the fact that most of the world is not yet vaccinated, strongly suggest that evidence-based communication campaigns promoting vaccine acceptability should be designed and disseminated. Building vaccination trust among university students through the spread of clear messages is key to the success of vaccinating many.

## Data Availability

The datasets used and/or analysed during the current study are available from the corresponding author on reasonable request.
